# Hypertrophy of the Gums

**Published:** 1856-10

**Authors:** Charles Bonsall

**Affiliations:** Cincinnati


### HYPERTROPHY OF THE GUMS.

MESSRS. EDITORS :—As you have requested some account of a case of Hypertrophy of the gums, which occurred in my practice last March, and to which Dr. Taylor referred in the April No. of the Register, I will now try to give you a short account of the case :

On the 5th of March, Miss B—— ——, aged about twenty-two, called on me to consult me as to the removal of a tumor from the inferior maxilla of the right side, which had recently shown indications of a more rapid growth than formerly, and on that account had caused her alarm.

Upon enquiry, I found that it had commenced several years previous, probably from some slight injury to the alveolus, between the cuspidati and the first bicuspid, and had gradually increased till the two teeth back of it, and three in front, had been thrown considerably out of their proper position, as may be inferred from the fact that there was a space of more than $\frac{5}{8}$ of an inch between the cuspidati and the first bicuspid.

Being an interesting case, I made an engagement for her to come the next day, and invited several members of the dental and medical professions to be present, at which time Drs. Taylor and Watt with myself, of the dental profession, and Prof. Judkins, an eminent surgeon, examined the tumor carefully, and decided unanimously that it was of an osteo-sarcomatous character, though not strictly belonging to that class of tumors, and that it was important that at least three teeth should be first removed (one having been extracted before I saw her) to insure the success of the operation.

The patient became alarmed and declined being operated on at this time, but on the 14th of March she came with a lady friend, and I removed the central incisor, cuspidati, and first bicuspid (the lateral incisor being already gone) and cut down to the bone all around the tumor, going a line or two beyond it, and then cut off, also, most of the alveolus down to nearly the lower line of the bottom of the sockets. I administered no anæsthetic, and the operation was not very painful, nor the flow of blood troublesome, so that the use of the cautery was unnecessary. From this time to the 10th of April, I occasionally touched some spot that showed the appearance of disease, with lunar caustic, till finally by the 20th of April the whole surface was entirely healed and looking healthy, and about May 20th I inserted a plate with three teeth on, which has been worn ever since, and there is at this time no appearance of any return of the tumor.

I preserved a cast of the case, taken before the operation, and one taken two months afterward, to deposit in the dental college collection.

Yours, truly,

CHARLES BONSALL.

*Cincinnati, Sept. 6th,* 1856.

